# The epidemiology of gonorrhoea in Norway, 1993–2007: past victories, future challenges

**DOI:** 10.1186/1471-2334-9-33

**Published:** 2009-03-19

**Authors:** Irena Jakopanec, Katrine Borgen, Preben Aavitsland

**Affiliations:** 1 Department of Infectious Disease Epidemiology, Norwegian Institute of Public Health, PO Box 4404 Nydalen, N-0403 Oslo, Norway

## Abstract

**Background:**

Gonorrhoea, a bacterial infection caused by *Neisseria gonorrhoeae*, has been increasing in several European countries, particularly among men who have sex with men (MSM) and teenagers. We describe the epidemiology of gonorrhoea in Norway in the recent 15 years in order to guide recommendations on the diagnosis, treatment and prevention of gonorrhoea. An evaluation of the Norwegian Surveillance System for Communicable Diseases (MSIS) in 1994, involving GPs and microbiological laboratories, suggested that the system has a high coverage, capturing over 90% of patients diagnosed with gonorrhoea.

**Methods:**

Using MSIS data on gonorrhoea cases we analysed specific trends by route of transmission, age, gender, anatomical sampling site, antimicrobial resistance and travel history from 1993–2007 and, to focus on more recent trends, from 2003–2007. MSM and heterosexual cases were defined by route of transmission.

**Results:**

From 1993 to 2007, 3601 gonorrhoea cases were reported. MSM cases increased from 10 in 1994 to 109 cases in 2004. From 2003–2007, the incidence of gonorrhoea was 5.4/100,000 person-years (95%CI: 4.9–6.0). Over these five years, MSM accounted for an average of 80 cases per year, of which 69% were infected by casual partners. In the same period, 98% of heterosexually infected had a positive swab from urethra only and only two (0.3%) from the pharynx. Only one woman (0.5%) was positive from the rectum. From 1993 – 2007, antimicrobial resistance results were reported for 3325 *N. gonorrhoeae *isolates (98% of cultured samples). The proportion resistant to quinolone has risen from 3% in 1995 to 47% in 2007, with 81% of the latter isolated from patients infected in Asia.

**Conclusion:**

The overall incidence of gonorrhoea in Norway remains low, but the increasing number of MSM cases calls for new, more effective approaches to prevention. Infections originating from abroad represent a constant risk of importing antimicrobial resistant *N. gonorrhoeae*. Due to the prevalence of quinolone resistant *N. gonorrhoeae *in Norway, third-generation cephalosporins should replace quinolones as the first choice in treatment guidelines. We advocate antimicrobial susceptibility testing for all cases and recommend taking samples for culture from all exposed anatomical sites.

## Background

Gonorrhoea, a bacterial infection caused by *Neisseria gonorrhoeae*, is a highly communicable [1] sexually transmitted infection (STI) and, due to a short incubation period, may serve as an indicator of recent risky sexual behaviour in symptomatic cases [2]. Since the seventies, when gonorrhoea was at its peak, the number of cases has decreased dramatically in many European countries [3,4]. Nevertheless, this preventable and treatable infection has been reported to be on the rise in several European countries [3,5] since the late nineties, particularly among men who have sex with men (MSM) [6-10] and teenagers [3,6,8].

Patients with gonorrhoea may experience symptoms such as purulent discharge and dysuria. The majority of heterosexual men (95% and more) are symptomatic, whereas up to 60% of women may be asymptomatic carriers of the disease for as long as 12 months [1]. Pharyngeal and rectal infections, which are mostly asymptomatic [1], may be important in gonorrhoeal transmission among MSM [11]. The risk of acquiring pharyngeal gonorrhoea by oral-genital heterosexual contact has been reported to be 14% for men and 31% for women [12]. Clinical trials reported pharyngeal gonorrhoea to be self-limiting within three months [13,14]. Untreated genital gonorrhoea may lead to serious late complications such as pelvic inflammatory disease, fistula formation and urethral strictures [1]. Furthermore, gonorrhoea increases susceptibility to HIV and HIV shedding in HIV positive patients [15].

Since the mid seventies, when penicillinase producing *N. gonorrhoeae *(PPNG) was first reported [1], treating gonorrhoea has presented an ongoing challenge around the world. In a sentinel surveillance study from 2004, significant proportions of *N. gonorrhoeae *isolates from 12 Western European countries were resistant to azithromycin, ciprofloxacin, penicillin or tetracycline and as much as 22% were resistant to more than one of these antimicrobials [16].

In Norway, under the Infectious Disease Control Act, all clinicians and laboratories are legally obliged to notify gonorrhoea cases to the Norwegian Institute of Public Health (NIPH). Using data from the Norwegian Surveillance System for Communicable Diseases (MSIS), we describe the epidemiology of gonorrhoea in Norway in the last 15 years (including specific trends by route of transmission, age, gender, antimicrobial resistance, place of infection and anatomical sampling sites) in order to develop targeted recommendations for the diagnosis, prevention and treatment of gonorrhoea [17].

## Methods

Cases fulfilling any of the following criteria should be reported to MSIS: 1. the patient has clinical symptoms compatible with gonorrhoea and is epidemiologically linked to another case; 2. *N. gonorrhoeae *was proven in the patient's sample by culture, antigen testing or nucleic acid amplification technique (NAAT) or 3. direct Gram-stained smear for microscopy shows intracellular diplococci. The case definition did not change during the study period. All clinicians and all of the approximately 20 clinical microbiology laboratories in Norway report to the system [18]. The Norwegian population during the study period was approximately 4.5 million.

Upon confirmation of a case of gonorrhoea, the laboratory sends a notification to the NIPH and a blank reporting form to the patient's clinician. The clinician fills the form with additional clinical and epidemiological data about the patient and sends it to the NIPH. All reports are anonymous and linked with a unique non-identifying number. NIPH uses laboratory reports to identify and remind clinicians if they fail to report on a case. The system achieves coverage of about 90% [18,19] and the data is of high quality; missing variables are rare. Most of the cases are initially reported by the laboratories; however, a minority of cases is reported directly from two venereal disease clinics and clinicians if direct microscopy of Gram-stained smear is used for establishing the diagnosis.

Among the key data collected by the surveillance system are: sex of the patient, date of sampling, month and year of birth, country of residence, country of birth, country of infection, reporting laboratory, type of diagnostic test used, anatomical sampling site, susceptibility of *N. gonorrhoeae *to antimicrobials, reasons for testing, transmission route and relation to the source person.

For the purpose of this study, we defined MSM cases as men who acquired gonorrhoea infection from another man (homosexual transmission). Similarly, we defined heterosexual cases as persons who acquired gonorrhoea infection from a partner of the opposite sex.

Reporting of PPNG was introduced in MSIS in 1993, while reporting of quinolone resistance started in 1995. No other resistance is currently reported in MSIS. Laboratories test all strains for penicillinase (betalactamase) production and for susceptibility to the most relevant antimicrobials, but the methods may vary by laboratory.

We analysed the data on all cases reported to MSIS from 1993 to 2007 by using Microsoft Excel and Stata 9.0. To describe more recent trends, we analysed data over a five year period from 2003 to 2007. We described cases according to demographic characteristics and various risk factors, including self-reported travel history and transmission route. The annual incidence with a 95% confidence interval (CI) was calculated using yearly population estimates by Statistics Norway http://www.ssb.no. We used Prais-Winsten autoregression to evaluate linear trends in all studied data over time, taking into account autocorrelation.

## Results

**Figure 1:**
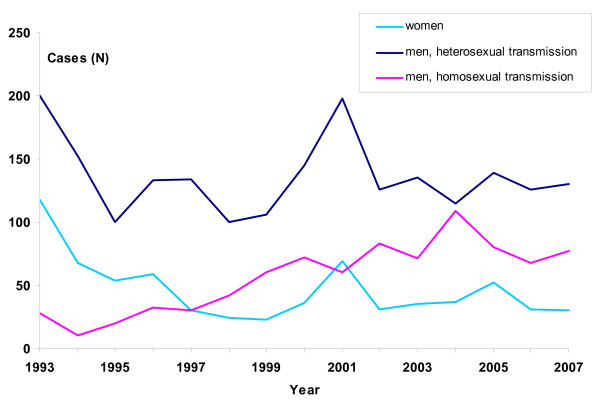
**Number of gonorrhoea cases by major transmission routes in Norway (N = 3578), 1993–2007**.

From 1993 to 2007 NIPH received 3601 reports of gonorrhoea cases diagnosed in Norway (Figure 1). The number of diagnoses decreased from a high in 1993 of 346 cases (8.0 per 100,000 population) to a low in 1998 of 166 cases (3.8 per 100,000 population). Cases peaked again in 2001 with 327 reports, of which 290 (89%) were diagnosed by culture and 35 (11%) by the newly available NAAT. The mean incidence in the recent five-year period (2003–2007) was 5.4 per 100,000 person-years (95% CI: 4.9–6.0). The sexual transmission route was reported for 3578 (99.4%) cases from 2003 to 2007 (Table 1).

**Table 1:** Selected characteristics of sexually infected gonorrhoea cases reported to the Norwegian surveillance system for communicable diseases (N = 1257), 2003–2007.

Characteristic	Selected categories	Sexual transmission route			
		
		Heterosexual		Homosexual	Unspecified
		
		Women n = 185 (%)	Men n = 645 (%)	Men n = 405 (%)	Men n = 22
Age	Median age in years	27	34	31	41
	
	10–19 years	32 (17)	25 (4)	20 (5)	0
	
	20–24 years	42 (23)	80 (12)	64 (16)	1
	
	25–34 years	59 (32)	216 (33)	160 (39)	5
	
	35–44 years	33 (18)	188 (29)	110 (27)	7
	
	≥ 45 years	19 (10)	136 (21)	51 (13)	9

Residence	Oslo city	64 (35)	229 (35)	310 (76)	13
	
	Other	121 (65)	416 (65)	95 (23)	9

Origin by birthplace	Norwegian	131 (71)	505 (78)	350 (86)	21
	
	European, other	16 (9)	51 (8)	32 (8)	0
	
	Asian	23 (12)	60 (9)	8 (2)	0
	
	African	6 (3)	22 (3)	5 (1)	1
	
	Other	9 (5)	7	10 (2)	0

Reason for visiting Norway	Temporary visit to Norway	15 (8)	19 (3)	13 (3)	0
	
	First generation immigrant	16 (9)	65 (10)	22 (5)	1
	
	Other, including permanent residents	154 (84)	561 (87)	370 (91)	21

Place of infection	Infected abroad	55 (30)	364 (56)	62 (15)	2
	
	- In Thailand	14 (8)	155 (24)	3 (7)	0
	
	Infected in Norway	124 (67)	274 (43)	341 (84)	15
	
	- In Oslo	47 (2)	145 (22)	294 (73)	13
	
	Unknown	6 (3)	7 (1)	2 (0.5)	5

Source partner	Steady partner	74 (40)	90 (14)	81 (20)	3
	
	Casual partner	84 (45)	388 (60)	280 (69)	4
	
	Prostitute	0	109 (17)	1 (0.2)	0
	
	Other	8 (4)	14 (2)	19 (5)	0
	
	Unknown	18 (10)	44 (7)	24 (6)	15

Indications for testing	Symptoms	114 (62)	631 (98)	331 (82)	20
	
	Contact tracing	34 (18)	10 (1)	26 (6)	0
	
	Own request	14 (8)	2 (0.3)	13 (3)	0
	
	Blood donor	1 (0.5)	1 (0.2)	0	0
	
	Pregnancy	1 (0.5)	/	/	/
	
	No specific reason	21 (11)	1 (0.2)	35 (8)	2

Diagnosed by	General practitioner, private specialist	90 (49)	384 (59)	119 (29)	21
	
	STI clinic	51 (28)	215 (33)	267 (66)	0
	
	Hospital	24 (13)	13 (2)	5 (1)	0
	
	Youth clinic	12 (6)	15 (2)	6 (1)	0
	
	Other	8 (4)	18 (3)	8 (2)	1

### Demographic data and groups by transmission route

**Figure 2:**
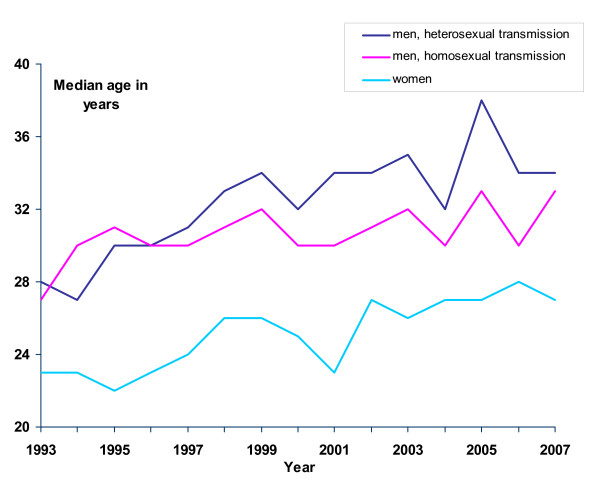
**Median age of patients with gonorrhoea in Norway by major transmission routes (N = 3578), 1993–2007**.

In the years 2003 to 2007, among teenagers (10 to19 years) females represented the majority of heterosexually transmitted cases (Table 1). Between 1993 and 2007, the number of cases among all teenage cases did not increase significantly (p = 0.100); however we did observe a concurrent increase among those aged 45 years and older in all transmission groups (p = 0.001), from 13 cases in 1993 to 48 cases in 2007. The median age among heterosexually infected men and women has increased since the early nineties (p < 0.001 for both), while the median age of MSM has remained relatively stable at around 29 years (p = 0.043) (Figure 2).

The majority of cases occurred in those born in Norway, although cases among migrants and visitors from other European countries and Asia are represented, especially among heterosexuals (Table 1).

In the period from 1993 to 2007, 842 MSM were diagnosed with gonorrhoea; from a low of 10 (4% of all cases) in 1994 to a high of 109 cases (41% of all cases) in 2004 (Figure 1). We observed a linear increase in the number of cases (p < 0.001) in this group during the study period, with an average of 80 cases per year since 2003. The majority resided in Oslo city and acquired their infection there (Table 1). In these five years, the proportion infected by casual partners varied from 60 to 80%.

**Figure 3:**
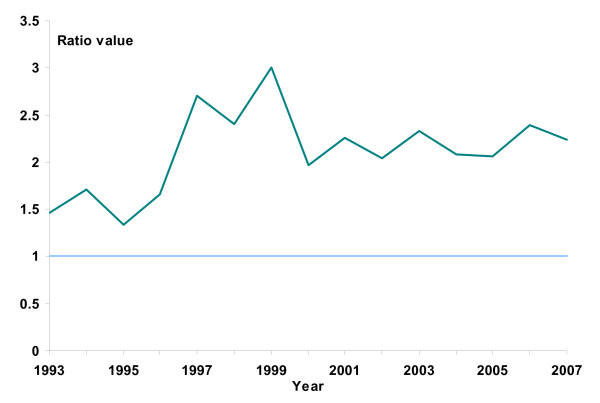
**Ratio of heterosexual men to heterosexual women, infected with gonorrhoea; domestic cases only (N = 1637), Norway, 1993–2007**.

Since 1996, the ratio of heterosexual men to women, infected with gonorrhoea in Norway, remained above 2 (Figure 3). Since 1993, symptoms were a reason for testing in 61% of infected women compared to 93% in men. The median duration of symptoms before sampling was four days for men and eight days for women. Between 2003 and 2007, 40% of the women got infected by steady partners compared to 14% of heterosexual men (Table 1).

### Anatomical locations of positive samples

From 1993 to 2007, 2677 (75%) of all cases were positive from a urethral swab. Of 91 patients diagnosed from a pharyngeal swab, 70 (77%) were MSM, 13 were women and eight were heterosexually infected men. Of 190 cases diagnosed from a rectal swab, 171 (90%) were MSM and 19 were female; among the females four had samples positive both from rectal and endocervical swabs. The number of positive rectal swabs in women has declined to one or less per year since 2001. From 2003–2007, no heterosexually infected man had gonorrhoea confirmed from more than one anatomical site (Table 2).

**Table 2:** Anatomical sites of *N. gonorrhoeae *isolates, reported to the Norwegian surveillance system for communicable diseases (N = 1257), 2003–2007.

*N. gonorrhoea *isolated from*:	Sexual transmission route			
	
	Heterosexual		Homosexual	Unspecified
	
	Women n = 185 (%)	Men n = 645 (%)	Men n = 405 (%)	Men n = 22
Urethra	8 (4)	631 (98)	276 (68)	22

Cervix	143 (79)	/	/	0

Rectum	0	0	68 (17)	0

Pharynx	7 (4)	2 (0.3)	20 (5)	0

Urethra, rectum and pharynx	1 (0.5)	0	4 (1)	0

Urethra and pharynx	0	0	7 (2)	0

Rectum and pharynx	0	0	8 (2)	0

Rectum and urethra	0	0	15 (4)	0

Cervix and pharynx	1 (0.5)	/	/	0

Cervix and urethra	19 (10)	/	/	0

Other, unspecified	3 (2)	4 (0.6)	1 (0.2)	0

Unknown	3 (2)	8 (1)	6 (1)	0

* the categories are mutually exclusive

### Antimicrobial resistance

**Figure 4:**
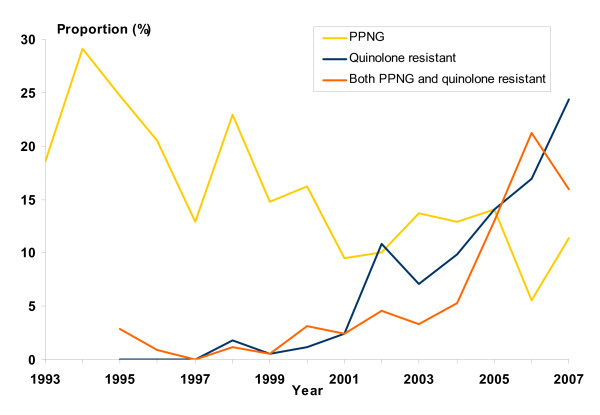
**Proportion of PPNG and quinolone resistance of 3399 cultured isolates of *N. gonorrhoeae *Norway, 1993–2007**. PPNG surveillance started in 1993 and quinolone surveillance in 1995.

From 1993 to 2007, 3399 cases (94%) were diagnosed by culture. In 2001, culturing was used in 89% of the cases, the lowest proportion in the entire period. Antimicrobial resistance was reported for 3325 (98%) of all cultured *N. gonorrhoeae *isolates. From 2003 to 2007, there has been a marked increase in the number of isolates reported to be quinolone resistant only, or both PPNG and quinolone resistant (Figure 4). Quinolone resistance is mainly found in isolates from patients infected in Asia. Among these patients, the proportion of quinolone resistance rose from 8% in 1998 to 81% in 2007.

### Imported gonorrhoea

In the early nineties, 70% of the cases reported acquiring their infection in Norway. This proportion decreased to around 60% in 2005 and 2006. Patients that reported unsafe sex during a recent travel to a foreign country prior to diagnosis, i.e. "imported gonorrhoea", were predominantly heterosexual men (80%). In 1993, 26% of the heterosexual infections among men were acquired outside Norway, compared to 62% in 2007. From 2003 to 2007, between 30 and 45 cases have been imported from Thailand to Norway every year. In 2003, 29% of all heterosexual male cases had been infected in Thailand. Other cases originated from around the world, with up to eight cases per year per country acquired in Pakistan, the Philippines, Brazil, Spain and Indonesia. Among those infected abroad, travellers aged 45 years and older represented 21% and those in the age group 35–44 years represented 30% in the period from 2003 to 2007.

## Discussion

The incidence of gonorrhoea in Norway from 2003 to 2007 (5.4 per 100,000 person-years) is similar to Sweden, Finland and Denmark and much lower than in the UK [20], making gonorrhoea a rare disease in Norway. However, several important challenges remain to be faced.

The majority of gonorrhoea cases are among heterosexual men, but because we lack a population denominator to calculate the incidence among MSM, it is not possible to conduct a proper comparison among the transmission groups. Nevertheless, we can conclude that MSM are currently the most vulnerable population to gonorrhoea infection in Norway and that preventive measures against STIs [21] among MSM are failing in Norway, similar to observations in other countries [8-10,22,23]. The rising trend of gonorrhoea among MSM is of particular concern as the proportion of cases infected through a casual partner is high, indicating increased risk for other STIs, including HIV. The majority of MSM get infected with gonorrhoea in the capital Oslo; therefore, preventive measures should be particularly focused on this area.

Among heterosexual cases, the median age is increasing. This may be due to the increasing age of the same risk group over time or the fading effect of preventive programmes primarily targeted at the young. Middle-aged men may be more able and willing to afford sex tourism in the areas of the world where gonorrhoea is still prevalent (see below). Based on similar observations of an increasing trend in reported gonorrhoea infections among people 45 years old and older, the need for interventions, aimed specifically at this group, has also been emphasised in the UK [24].

Among 21 European countries with diverse surveillance systems, Norway had the eighth highest men to women ratio of gonorrhoea cases in 2006 [20]. Among heterosexually acquired domestic cases, the number of males diagnosed is 2 to 3 times greater than females, similar to reports from other countries [6,9]. Reasons for this gender disparity may include: behaviour differences (promiscuity, visiting prostitutes, partner notification etc.), biological differences in developing symptoms, duration of infection and a pool of undiagnosed cases among asymptomatic women. Since women are more susceptible to infection [1] and frequently experience an asymptomatic course of infection, the persistence of endemic gonorrhoea in Norway might be fostered by undiagnosed women. The gender ratio implies that a more thorough approach to contact tracing is warranted; however this is limited by the frequency of infections in heterosexual men associated with casual partners or prostitutes (Table 1), making partner notification difficult. Since symptoms were stated as a reason for testing in only 60% of women, we can conclude that asymptomatic cases are being diagnosed with gonorrhoea as well.

While gonorrhoea has become rare in Norway, it should still be considered as a diagnostic option. When making decisions for testing, clinicians should be aware that many women in Norway, similar to reports from other countries [9,25], acquire their infection from a steady partner, having no obvious risk-factors for gonorrhoea in their medical history. Furthermore, belated diagnosis might lead to severe health complications as we experienced in a recent gonorrhoea outbreak in Norway in 2008 [26].

The number of samples tested with a negative result in Norway is unknown. There is little evidence whether sampling from several anatomic sites increases the diagnostic sensitivity [27], but studies among MSM show that sampling from the urethra only may lead to a significant proportion (up to 40%) of missed cases [22,28,29]. Regardless of the exposure, rectal co-infection with cervical gonorrhoea has been reported in up to 30% of infected women [1], therefore the low number of women who recently tested positive from the rectum (Table 2) may suggest that rectal samples from women are rarely taken. In a study in France, a prevalence of 6% of pharyngeal gonorrhoea among heterosexual men with urethral gonorrhoea has been reported from 1999 to 2001 [23]. To tackle the possible undiagnosed reservoir of infection, it is important that sampling is carried out according to the exposures rather than the presence of symptoms; although some patients might be reluctant to provide the details of their exposure (see "Unspecified" group, Table 2).

Antimicrobial resistant *N. gonorrhoeae *is an increasing problem in Norway, exacerbated by a large proportion of imported infections from Asia. Following their importation into Norway and subsequent onward spread within the population, infections originating abroad are not easily identifiable [26]. Recently reported travel is not a reliable tool to guide treatment choices. More than 40% of infections in 2006 and 2007 were quionolone resistant, regardless of whether they were acquired domestically or abroad (Figure 4), which implies that third generation cephalosporins should replace quinolones as the first choice empirical treatment for gonorrhoeal infections in the Norwegian treatment recommendations [30]; similar to recent recommendations in other countries [31,32]. As resistance to third generation cephalosporins is already emerging [16], improved surveillance of *N. gonorrhoeae *resistance, involving reporting resistance to any relevant antimicrobial, using nationally standardized methods, is necessary for the timely review and revision of national treatment guidelines. This may help to ensure that the most clinically effective empirical treatments, ideally achieving a cure rate of over 95% [33], will be used in the future. This is feasible in Norway due to the prevalent practice of diagnosing gonorrhoea with culture and a high coverage of reporting to MSIS.

Although travellers who got infected with gonorrhoea represent a diverse group, some studies identified demographic factors such as male sex, single status and age of <20 years [34] as associated with a higher frequency of casual sexual intercourse abroad, while others identified middle-aged and married travellers [35] as high-risk groups. It is therefore interesting to note that male travellers, older than 45 years, represent a significant proportion among our cases and that as much as half of the infected travellers are older than 34 years.

Among all the infections acquired outside of Norway, Thailand, a known sex tourism destination [34] remains the most prominent country associated with the acquisition of gonorrhoea since the nineties [36], especially among heterosexual men. A similar situation has been described in Denmark and Sweden [6,9]. Due to the high prevalence of HIV infection in Thailand, the rise of imported gonorrhoea is a stark reminder of the high-risk sexual behaviour of some Norwegian travellers. Asymptomatic travellers who had casual sex abroad rarely present at the doctor's office, therefore screening for STIs might not be possible [34].

We identified some potential limitations and weaknesses of our study. The evaluation of the STIs reporting coverage to MSIS was done more than a decade ago. All behavioural data in the system are self-reported. As the spectrum of collected variables in Norway is rather broad, some missing data were noted. We defined MSM and heterosexual cases according to the reported route of transmission. This definition provides no insight into the actual sexual orientation, behaviour or sexual practices of the patients and is only related to a single exposure, at which patients got infected. Culturing of *N. gonorrhoeae*, a method most frequently used for laboratory confirmation of gonorrhoea in Norway, has specificity of 99% and sensitivity of 60 to 70% [37], which might be further affected by transport conditions. Therefore, a negative laboratory sample does not exclude gonorrhoea. Patients with negative tests should still be reported, providing they experience clinical symptoms compatible with gonorrhoea and are epidemiologically linked to another case. In this scenario, reporting should arise from the clinicians' initiative; however no such cases were reported during the entire period from 1993 to 2007. This could indicate that a small proportion of gonorrhoea infections in Norway remain unreported. Although clinicians are strongly encouraged to obtain a sample for culturing, some may skip reporting of cases diagnosed with direct microscopy. Nevertheless, we consider MSIS a representative and reliable source of data on gonorrhoea cases in Norway.

The observed peak incidence in heterosexual cases in 2001 was partially influenced by the decision of one laboratory to screen samples collected for Chlamydia testing, with NAAT for both Chlamydia and gonorrhoea. In 2001, this laboratory reported 46 cases in total – much higher than in the previous (4 cases) and the following year (17 cases). Using NAAT for screening in low prevalence populations has been associated with lower positive predictive value [38] and some of the reported cases might have been false positive. This laboratory continued to use NAAT in the following years and reported it as the diagnostic method in 75% of cases. Nevertheless, the peak in 2001 remains prominent even after excluding the cases diagnosed with NAAT, and is largely due to heterosexual cases infected in Norway. No increase in HIV and syphilis was observed in this group at the same time or a year later [17].

## Conclusion

The overall incidence of gonorrhoea in Norway is low. Heterosexual transmission is fairly stable, while there is a worrisome increase among MSM. Since most of the MSM report getting infected in Norway (Oslo), prevention efforts at local MSM venues should continue. Further research is necessary to identify more effective prevention measures and reasons for the resurgence of STIs among MSM Europe-wide. Gonorrhoea is frequently brought to Norway from abroad with a higher risk of imported cases being resistant to antimicrobials. As the link to the foreign country might be lost soon after the introduction to Norway, the widespread practice of culturing which enables antimicrobial susceptibility testing should be further encouraged. There is a need for standardisation of national laboratory methods for susceptibility testing and for improving the surveillance of antimicrobial resistance to enable rapid revision of treatment guidelines when necessary. We recommend taking samples for culture of *N. gonorrhoeae *from all exposed anatomical sites.

## Competing interests

The authors declare that they have no competing interests.

## Authors' contributions

IJ performed the data analysis and drafted the manuscript, KB assisted in the interpretation of data and drafting of the manuscript, PA participated in data analysis and drafting of the manuscript. All authors read and approved the final manuscript.

## Pre-publication history

The pre-publication history for this paper can be accessed here:

http://www.biomedcentral.com/1471-2334/9/33/prepub
